# Supplementary material for: Paralytic Shellfish Toxins in Alaskan Butter Clams: Does Cleaning Make Them Safe to Eat?
Source: Toxins (Basel). 2025 May 28;17(6):271. doi: 10.3390/toxins17060271 (PMC12197486; doi:10.3390/toxins17060271)
Supplement: Supplementary file 1 [file toxins-17-00271-s001.zip › Supplementary Table S1.pdf]

Supplementary Table S1

Date Sampled	Replicate number	Number clams per batch	Black tip mass per batch clams (g)	Neck mass per batch clams (g)	Gut mass per batch clams (g)	Body mass per batch clams (g)	Total mass per batch clams (g)	Black tip µg STX-eq. / 100 g tissue from HPLC per batch clams	Neck µg STX-eq. / 100 g tissue from HPLC per batch clams	Gut µg STX-eq. / 100 g tissue from HPLC per batch clams	Body µg STX-eq. / 100 g tissue from HPLC per batch clams	Whole clams µg STX-eq. / 100 g tissue from HPLC per batch clams	Black tip µg STX-eq in tissue from HPLC per batch clams	Neck µg STX-eq. in tissue from HPLC per batch clams	Gut µg STX-eq. in tissue from HPLC per batch clams	Body µg STX-eq. in tissue from HPLC per batch clams
04/17/18	1	12	8.3	36.4	27.9	178.4	251.0	198.2	152.0	48.1	39.0	61.6	16.37	55.37	13.42	69.53
04/17/18	2	12	8.5	30.4	26.2	172.4	237.5	253.2	155.1	13.2	15.5	41.6	21.49	47.12	3.46	26.67
04/17/18	3	12	7.6	30.5	22.7	164.2	225.0	206.5	201.5	20.6	36.7	63.1	15.68	61.50	4.67	60.24
04/17/18	4	12	8.1	27.5	24.2	159.0	218.8	230.0	198.2	19.5	41.4	65.6	18.54	54.60	4.72	65.77
04/19/18	1	12	10.1	30.0	21.5	188.3	249.8	367.5	182.6	206.0	79.7	114.5	37.08	54.72	44.24	150.04
04/19/18	2	12	10.4	27.6	22.1	187.8	248.0	359.5	186.7	163.5	67.0	101.2	37.50	51.57	36.13	125.81
04/19/18	3	12	8.9	25.7	19.8	165.6	220.0	314.3	197.2	172.3	85.9	115.9	27.84	50.63	34.11	142.26
05/02/17	1	12	6.83	24.53	21.19	155.00	207.6	29.5	90.2	4.1	37.4	40.0	2.01	22.12	0.87	58.05
05/02/17	2	12	9.53	27.54	26.66	154.73	218.5	20.2	22.4	2.5	24.1	21.1	1.93	6.16	0.67	37.23
05/02/17	3	12	6.62	24.84	25.57	140.76	197.8	26.6	50.1	2.7	25.0	25.3	1.76	12.45	0.68	35.14
05/02/17	5	12	7.42	28.39	25.84	158.64	220.3	21.6	48.6	3.8	24.6	25.1	1.60	13.79	0.98	38.97
05/02/17	6	12	6.15	31.55	25.31	182.28	245.3	28.9	66.7	4.1	20.2	24.7	1.78	21.06	1.03	36.75
05/25/17	1	12	6.8	24.5	21.2	155.0	207.6	431.8	367.6	19.3	24.2	77.7	29.49	90.17	4.08	37.45
05/25/17	2	12	9.5	27.5	26.7	154.7	218.5	212.4	81.2	9.5	15.6	31.7	20.25	22.36	2.53	24.06
05/25/17	3	12	6.6	24.8	25.6	140.8	197.8	401.5	201.8	10.4	17.7	52.8	26.58	50.14	2.67	24.97
05/25/17	4	12	6.1	26.8	16.8	146.8	196.6	417.7	155.8	13.7	23.8	53.2	25.52	41.78	2.31	35.02
05/25/17	5	12	7.4	28.4	25.8	158.6	220.3	291.1	171.1	14.6	15.5	44.7	21.60	48.56	3.78	24.56
05/25/17	6	12	6.2	31.6	25.3	182.3	245.3	469.8	211.6	16.1	11.1	48.9	28.89	66.75	4.08	20.16
06/15/15	1	12	9.92	30.55	21.60	161.10	223.2	436.9	337.0	1556.2	20.5	231.0	43.34	102.95	336.13	33.08
06/15/15	2	12	11.0	30.1	17.4	174.9	233.3	605.7	184.3	668.8	789.0	693.4	66.32	55.44	116.38	1379.72
06/15/15	3	12	8.7	25.7	14.1	134.0	182.5	144.5	130.6	1912.9	334.2	418.3	12.63	33.59	269.53	447.74
06/15/15	4	12	8.9	28.4	16.6	156.9	210.9	428.3	247.9	462.2	80.6	147.9	38.29	70.34	76.82	126.52
06/09/16	1	11	7.3	19.1	15.2	123.3	164.9	199.8	1183.7	1230.6	326.8	504.0	14.51	226.21	187.29	402.85
06/09/16	2	11	6.6	18.3	15.1	92.6	132.7	563.5	295.9	1251.4	294.2	416.8	37.14	54.25	188.96	272.54
06/09/16	3	11	7.41	19.27	14.52	98.98	140.2	465.8	159.3	1166.0	349.1	413.8	34.52	30.70	169.30	345.55
06/09/16	4	10	6.3	22.4	14.6	89.3	132.7	439.2	167.0	1861.4	786.4	783.6	27.67	37.43	271.77	702.53
06/09/16	5	10	8.9	17.1	15.5	86.5	128.1	706.4	119.0	1241.0	276.1	401.9	62.73	20.38	192.73	238.93
06/22/17	1	12	5.3	20.4	13.7	122.3	161.7	119.0	101.7	49.0	16.4	33.3	6.32	20.76	6.72	20.11
06/22/17	2	11	5.8	21.3	14.7	132.5	174.4	106.2	102.9	49.5	17.8	33.8	6.15	21.94	7.30	23.60
06/22/17	3	12	6.2	22.5	16.8	141.1	186.5	123.5	70.8	44.6	19.5	31.4	7.63	15.91	7.48	27.47
06/22/17	4	12	6.3	22.9	15.7	125.6	170.5	166.6	97.2	40.7	19.2	37.1	10.49	22.21	6.39	24.08
06/22/17	5	12	6.3	22.7	18.6	120.6	168.1	107.0	99.6	17.9	14.8	30.0	6.74	22.56	3.34	17.80
06/22/17	6	11	5.3	21.0	14.4	108.7	149.4	129.3	96.3	40.7	13.7	32.0	6.84	20.22	5.88	14.88
6/18/2018	1	12	7.6	31.9	22.1	158.1	219.7	212.2	162.5	1937.3	142.9	328.6	16.21	51.83	428.14	225.90
6/18/2018	2	12	8.1	32.9	19.1	152.0	212.1	258.4	141.0	2003.6	144.7	315.4	21.04	46.42	381.69	219.94
6/18/2018	3	12	8.7	33.2	21.4	154.9	218.2	220.6	131.6	2491.8	151.3	380.9	19.26	43.68	533.75	234.39
7/7/2018	1	12	7.5	33.9	20.7	139.9	202.0	214.1	148.2	329.4	414.7	353.8	15.97	50.25	68.28	580.21
7/7/2018	2	12	7.4	33.5	19.7	150.2	210.7	198.4	88.6	315.4	320.2	278.7	14.68	29.64	62.07	480.88
7/7/2018	3	12	6.1	26.3	16.7	138.3	187.4	212.4	138.7	349.7	319.9	293.6	12.91	36.46	58.50	442.36

Date Sampled	Replicate number	Number clams per batch	Whole clam minus viscera (µg STX-eq. / 100 g tissue)	Whole clam minus viscera and back tip (µg STX-eq. / 100 g tissue)	Whole clam minus siphon (µg STX-eq. / 100 g tissue)	Clam body only (µg STX-eq. / 100 g tissue)	Whole clam µg STX-eq in 200 g meal	Whole clam minus viscera µg STX-eq in 200 g meal	Whole clam minus viscera and back tip µg STX-eq in 200 g meal	Whole clam minus siphon in 200 g meal	Clam body only µg STX-eq in 200 g meal	Reduction in the toxicity of clams minus viscera relative to whole clams (%)	Reduction in the toxicity of clams minus viscera and back tip relative to whole clams (%)	Reduction in the toxicity of clams minus siphon relative to whole clams (%)	Reduction in the calm body only relative to whole clams (%)	City	Collection site
04/17/18	1	12	63.3	58.2	46.0	39.0	123.3	126.7	116.3	92.1	78.0	-2.8	5.7	25.3	36.8	Kodiak	Mission Beach
04/17/18	2	12	45.1	36.4	26.6	15.5	83.2	90.2	72.8	53.2	30.9	-8.4	12.5	36.0	62.8	Kodiak	Mission Beach
04/17/18	3	12	67.9	62.5	44.2	36.7	126.3	135.8	125.0	88.4	73.4	-7.6	1.0	30.0	41.9	Kodiak	Mission Beach
04/17/18	4	12	71.4	64.5	50.5	41.4	131.3	142.8	129.1	100.9	82.7	-8.8	1.7	23.1	37.0	Kodiak	Mission Beach
04/19/18	1	12	105.9	93.8	94.3	79.7	229.0	211.8	187.6	188.6	159.4	7.5	18.1	17.6	30.4	Old Harbor	Shipwreck Beach
04/19/18	2	12	95.1	82.3	82.4	67.0	202.5	190.3	164.7	164.8	134.0	6.0	18.7	18.6	33.8	Old Harbor	Shipwreck Beach
04/19/18	3	12	110.3	100.8	97.5	85.9	231.7	220.6	201.7	195.0	171.8	4.8	13.0	15.9	25.9	Old Harbor	Shipwreck Beach
05/02/17	1	12	44.1	44.7	37.1	37.4	80.0	88.2	89.3	74.2	74.9	-10.2	-11.6	7.2	6.4	Kodiak	Mission Beach
05/02/17	2	12	23.6	23.8	23.8	24.1	42.1	47.3	47.6	47.7	48.1	-12.2	-13.1	-13.2	-14.3	Kodiak	Mission Beach
05/02/17	3	12	28.7	28.7	25.0	25.0	50.6	57.3	57.5	50.1	49.9	-13.3	-13.6	1.0	1.3	Kodiak	Mission Beach
05/02/17	5	12	28.0	28.2	24.4	24.6	50.2	55.9	56.4	48.9	49.1	-11.3	-12.3	2.7	2.2	Kodiak	Mission Beach
05/02/17	6	12	27.1	27.0	20.4	20.2	49.4	54.2	54.1	40.9	40.3	-9.6	-9.4	17.3	18.4	Kodiak	Mission Beach
05/25/17	1	12	84.3	71.1	41.4	24.2	155.3	168.6	142.2	82.7	48.3	-8.5	8.5	46.7	68.9	Old Harbor	Shipwreck Beach
05/25/17	2	12	34.8	25.5	27.0	15.6	63.4	69.5	50.9	54.0	31.1	-9.7	19.6	14.8	50.9	Old Harbor	Shipwreck Beach
05/25/17	3	12	59.0	45.4	35.0	17.7	105.5	118.1	90.7	69.9	35.5	-11.9	14.0	33.7	66.4	Old Harbor	Shipwreck Beach
05/25/17	4	12	56.9	44.2	39.6	23.8	106.5	113.8	88.5	79.2	47.7	-6.9	16.9	25.6	55.2	Old Harbor	Shipwreck Beach
05/25/17	5	12	48.7	39.1	27.8	15.5	89.4	97.4	78.2	55.6	31.0	-8.9	12.6	37.8	65.4	Old Harbor	Shipwreck Beach
05/25/17	6	12	52.6	40.6	26.0	11.1	97.7	105.3	81.3	52.1	22.1	-7.7	16.8	46.7	77.4	Old Harbor	Shipwreck Beach
06/15/15	1	12	89.0	71.0	44.7	20.5	462.0	178.0	142.0	89.4	41.1	61.5	69.3	80.7	91.1	Old Harbor	Shipwreck Beach
06/15/15	2	12	695.4	700.2	778.2	789.0	1386.9	1390.8	1400.4	1556.3	1577.9	-0.3	-1.0	-12.2	-13.8	Old Harbor	Shipwreck Beach
06/15/15	3	12	293.3	301.4	322.6	334.2	836.6	586.5	602.8	645.1	668.4	29.9	27.9	22.9	20.1	Kodiak	Shipwreck Beach
06/15/15	4	12	121.0	106.2	99.4	80.6	295.9	242.1	212.5	198.7	161.2	18.2	28.2	32.8	45.5	Kodiak	Shipwreck Beach
06/09/16	1	11	430.1	441.8	319.8	326.8	1008.0	860.2	883.7	639.5	653.7	14.7	12.3	36.6	35.2	Kodiak	Mission Beach
06/09/16	2	11	309.6	294.5	312.1	294.2	833.6	619.2	589.0	624.2	588.4	25.7	29.3	25.1	29.4	Kodiak	Mission Beach
06/09/16	3	11	326.9	318.2	357.2	349.1	827.6	653.8	636.4	714.5	698.2	21.0	23.1	13.7	15.6	Kodiak	Mission Beach
06/09/16	4	10	650.3	662.2	763.5	786.4	1567.1	1300.5	1324.3	1527.0	1572.7	17.0	15.5	2.6	-0.4	Kodiak	Mission Beach
06/09/16	5	10	286.2	250.2	316.2	276.1	803.9	572.3	500.3	632.4	552.3	28.8	37.8	21.3	31.3	Kodiak	Mission Beach
06/22/17	1	12	31.9	28.6	20.7	16.4	66.7	63.8	57.3	41.4	32.9	4.4	14.1	37.9	50.7	Kodiak	Mission Beach
06/22/17	2	11	32.4	29.6	21.5	17.8	67.7	64.8	59.2	43.0	35.6	4.3	12.5	36.4	47.4	Kodiak	Mission Beach
06/22/17	3	12	30.1	26.5	23.8	19.5	62.7	60.1	53.1	47.7	39.0	4.2	15.4	24.0	37.9	Kodiak	Mission Beach
06/22/17	4	12	36.7	31.2	26.2	19.2	74.1	73.4	62.4	52.4	38.3	1.0	15.9	29.3	48.3	Kodiak	Mission Beach
06/22/17	5	12	31.5	28.2	19.3	14.8	60.0	63.0	56.4	38.7	29.5	-5.0	6.1	35.5	50.8	Kodiak	Mission Beach
06/22/17	6	11	31.1	27.1	19.1	13.7	64.0	62.2	54.1	38.1	27.4	2.9	15.4	40.5	57.2	Kodiak	Mission Beach
6/18/2018	1	12	148.7	146.2	146.1	142.9	657.3	297.5	292.4	292.2	285.8	54.7	55.5	55.5	56.5	Kodiak	Mission Beach
6/18/2018	2	12	148.9	144.0	150.5	144.7	630.9	297.7	288.1	301.0	289.4	52.8	54.3	52.3	54.1	Kodiak	Mission Beach
6/18/2018	3	12	151.1	147.9	155.0	151.3	761.7	302.2	295.7	310.1	302.7	60.3	61.2	59.3	60.3	Kodiak	Mission Beach
7/7/2018	1	12	356.6	362.7	404.5	414.7	707.6	713.2	725.4	809.1	829.4	-0.8	-2.5	-14.3	-17.2	Kodiak	Mission Beach
7/7/2018	2	12	274.9	278.0	314.5	320.2	557.4	549.9	556.0	629.0	640.4	1.4	0.2	-12.8	-14.9	Kodiak	Mission Beach
7/7/2018	3	12	288.2	291.0	315.4	319.9	587.3	576.3	581.9	630.7	639.8	1.9	0.9	-7.4	-8.9	Kodiak	Mission Beach

Table S1. Data on how different cleaning methods impacts concentration of STX-eq. in edible tissues based on data from the 2015 to 2018 study in Kodiak Alaska [11].
