# Supplementary material for: Paralytic Shellfish Toxins in Alaskan Butter Clams: Does Cleaning Make Them Safe to Eat?
Source: Toxins (Basel). 2025 May 28;17(6):271. doi: 10.3390/toxins17060271 (PMC12197486; doi:10.3390/toxins17060271)
Supplement: Supplementary file 1 [file toxins-17-00271-s001.zip › Supplementary Table S2.pdf]

Supplementary Table S2

**1967 Neal dissertation**	**Latitude**	**Longitude**
Ham Island Passage	55.193056	-131.762222
Mud Bay	55.413889	-131.765278
Ryus Bay	54.946389	-131.401111
Vallenar Rock station	55.425000	-131.856944

**Chambers & Magnusson 1950**		
Ham Island	55.198333	-131.361110
Ham Island	55.196853	-131.386389
Annette Island	55.196389	-131.388889
Dall Bay	55.150556	-131.731944
Dall Bay	55.150556	-131.732500
Carlton Island	55.948611	-132.366667
Carlton Island	55.913889	-132.363889
Carlton Island	55.900000	-132.380556
Petersburg	56.815460	-132.955940
Pybus Bay	57.315200	-134.093592
Roberts Island	57.301389	-133.463889
Roberts Island	57.266667	-133.458333
Fanshaw	57.216667	-133.500000
Chaik Bay	57.316389	-134.563889
Chaik Bay	57.331944	-134.563889
Hood Bay	57.443056	-134.536111
Hood Bay	57.443056	-134.552778
Hood Bay	57.448611	-134.548611
Security Bay	56.845833	-134.336111
Security Bay	56.863889	-134.351389

Table S2. Sampling locations for determining toxicity in whole clams from Chambers & Magnusson (1950) [9] and Neal (1967) [10].
