# Supplementary material for: Paralytic Shellfish Toxins in Alaskan Butter Clams: Does Cleaning Make Them Safe to Eat?
Source: Toxins (Basel). 2025 May 28;17(6):271. doi: 10.3390/toxins17060271 (PMC12197486; doi:10.3390/toxins17060271)
Supplement: Supplementary file 1 [file toxins-17-00271-s001.zip › Supplementary Table S3.pdf]

Supplementary Table 3

Date	Detectable Toxin	Mouse units per 100 g tissue	µg STX-eq. per 100 g tissue
3/1/1948	Y	1394	279
5/1/1948	Y	1160	232
5/1/1948	Y	880	176
5/1/1948	Y	1520	304
5/1/1948	Y	1120	224
5/1/1948	Y	920	184
5/1/1948	Y	260	52
5/1/1948	Y	660	132
5/1/1948	Y	640	128
5/1/1948	Y	76	15
5/1/1948	Y	1620	324
5/1/1948	Y	1600	320
5/1/1948	Y	628	126
5/1/1948	Y	462	92
5/1/1948	Y	1960	392
5/1/1948	Y	2120	424
5/1/1948	Y	828	166
5/1/1948	Y	1678	336
7/1/1948	Y	5080	1016
7/1/1948	Y	3880	776
7/1/1948	Y	5160	1032
7/1/1948	Y	3820	764
7/1/1948	Y	3240	648
7/1/1948	Y	1440	288
7/1/1948	Y	810	162
7/1/1948	Y	700	140
7/1/1948	Y	40	8
7/1/1948	Y	2060	412
7/1/1948	Y	3060	612
7/1/1948	Y	404	81
7/1/1948	Y	3760	752
7/1/1948	Y	1500	300
7/1/1948	Y	1460	292
7/1/1948	Y	2752	550
7/1/1948	Y	2270	454
7/1/1948	Y	3222	644

7/1/1948	Y	1446	289
7/1/1948	Y	1074	215
8/1/1948	Y	6360	1272
8/1/1948	Y	3758	752
8/1/1948	Y	5320	1064
8/1/1948	Y	3320	664
8/1/1948	Y	2520	504
8/1/1948	Y	940	188
8/1/1948	Y	810	162
8/1/1948	Y	986	197
8/1/1948	Y	46	9
8/1/1948	Y	4386	877
8/1/1948	Y	3080	616
8/1/1948	Y	3020	604
8/1/1948	Y	520	104
8/1/1948	Y	1600	320
8/1/1948	Y	480	96
8/1/1948	Y	4760	952
8/1/1948	Y	2924	585
8/1/1948	Y	1908	382
8/1/1948	Y	1606	321
8/1/1948	Y	1494	299
8/1/1948	Y	1236	247
8/1/1948	Y	1944	389
9/1/1948	Y	1500	300
9/1/1948	Y	940	188
9/1/1948	Y	826	165
9/1/1948	Y	42	8
9/1/1948	Y	2592	518
9/1/1948	Y	460	92
9/1/1948	Y	3240	648
9/1/1948	Y	392	78
9/1/1948	Y	3640	728
9/1/1948	Y	2780	556
9/1/1948	Y	3588	718
9/1/1948	Y	1512	302
9/1/1948	Y	362	72
9/1/1948	Y	1160	232
9/1/1948	Y	1070	214
9/1/1948	Y	1472	294
9/1/1948	Y	2702	540

9/30/1948	Y	2335	467
9/30/1948	Y	4123	825
9/30/1948	Y	5691	1138
9/30/1948	Y	4096	819
9/30/1948	Y	3875	775
9/30/1948	Y	2106	421
9/30/1948	Y	2590	518
9/30/1948	Y	1612	322
9/30/1948	Y	1218	244
9/30/1948	Y	2987	597
9/30/1948	Y	4100	820
10/1/1948	Y	1520	304
10/1/1948	Y	1460	292
10/1/1948	Y	1380	276
10/1/1948	Y	1520	304
10/1/1948	Y	1140	228
10/1/1948	Y	156	31
10/1/1948	Y	44	9
10/1/1948	Y	4020	804
10/1/1948	Y	3000	600
10/1/1948	Y	3660	732
10/1/1948	Y	3940	788
10/1/1948	Y	3980	796
10/1/1948	Y	2920	584
10/1/1948	Y	2952	590
10/1/1948	Y	1260	252
10/1/1948	Y	2120	424
10/1/1948	Y	1698	340
10/1/1948	Y	1832	366
10/1/1948	Y	2820	564
10/1/1948	Y	2800	560
11/1/1948	Y	1420	284
11/1/1948	Y	3920	784
11/1/1948	Y	454	91
11/1/1948	Y	414	83
11/1/1948	Y	584	117
11/1/1948	Y	42	8
11/1/1948	Y	2352	470
11/1/1948	Y	2168	434
11/1/1948	Y	2386	477
11/1/1948	Y	3900	780

11/1/1948	Y	2080	416
11/1/1948	Y	1162	232
11/1/1948	Y	1320	264
12/1/1948	Y	2680	536
12/1/1948	Y	1344	269
12/1/1948	Y	1536	307
12/1/1948	Y	4320	864
12/1/1948	Y	3080	616
12/1/1948	Y	1340	268
12/1/1948	Y	1060	212
12/1/1948	Y	1154	231
1/1/1949	Y	4120	824
1/1/1949	Y	2310	462
1/1/1949	Y	3800	760
1/1/1949	Y	3260	652
1/1/1949	Y	3020	604
1/1/1949	Y	42	8
2/1/1949	Y	5100	1020
2/1/1949	Y	2600	520
2/1/1949	Y	3620	724
3/1/1949	Y	4600	920
3/1/1949	Y	1800	360
3/1/1949	Y	4040	808
3/1/1949	Y	1234	247
3/1/1949	Y	1100	220
3/1/1949	Y	770	154
3/1/1949	Y	40	8
3/1/1949	Y	2820	564
3/1/1949	Y	1920	384
3/1/1949	Y	2660	532
3/1/1949	Y	3320	664
3/1/1949	Y	3720	744
3/1/1949	Y	2128	426
3/1/1949	Y	1814	363
3/1/1949	Y	2420	484
3/1/1949	Y	936	187
3/1/1949	Y	1032	206
3/1/1949	Y	1998	400
4/1/1949	Y	4840	968
4/1/1949	Y	2334	467
4/1/1949	Y	3060	612

4/1/1949	Y	2520	504
4/1/1949	Y	2680	536
4/1/1949	Y	1100	220
4/1/1949	Y	1220	244
4/1/1949	Y	940	188
4/1/1949	Y	40	8
4/1/1949	Y	3500	700
4/1/1949	Y	2320	464
4/1/1949	Y	2384	477
4/1/1949	Y	3040	608
4/1/1949	Y	3240	648
4/1/1949	Y	360	72
4/1/1949	Y	278	56
4/1/1949	Y	768	154
4/1/1949	Y	1312	262
4/1/1949	Y	1410	282
4/4/1949	Y	1200	240
4/4/1949	Y	1688	338
5/1/1949	Y	4600	920
5/1/1949	Y	2420	484
5/1/1949	Y	3680	736
5/1/1949	Y	3300	660
5/1/1949	Y	2760	552
5/1/1949	Y	774	155
5/1/1949	Y	966	193
5/1/1949	Y	878	176
5/1/1949	N	0	0
5/1/1949	Y	3560	712
5/1/1949	Y	2260	452
5/1/1949	Y	3080	616
5/1/1949	Y	500	100
5/1/1949	Y	2300	460
5/1/1949	Y	1778	356
5/1/1949	Y	1480	296
5/1/1949	Y	2036	407
5/1/1949	Y	972	194
5/1/1949	Y	948	190
6/1/1949	Y	1520	304
6/1/1949	Y	1340	268
6/1/1949	Y	880	176
6/1/1949	N	0	0

6/1/1949	Y	2680	536
6/1/1949	Y	2100	420
6/1/1949	Y	3260	652
6/1/1949	Y	2380	476
6/1/1949	Y	660	132
6/1/1949	Y	2370	474
6/1/1949	Y	1692	338
6/1/1949	Y	2080	416
6/1/1949	Y	1114	223
6/1/1949	Y	1982	396
7/1/1949	Y	3280	656
7/1/1949	Y	1244	249
7/1/1949	Y	2500	500
7/1/1949	Y	1380	276
7/1/1949	Y	1820	364
7/1/1949	Y	1800	360
7/1/1949	N	0	0
7/1/1949	Y	2200	440
7/1/1949	Y	2220	444
7/1/1949	Y	3440	688
7/1/1949	Y	3340	668
7/1/1949	Y	3300	660
7/1/1949	Y	3500	700
7/1/1949	Y	2160	432
7/1/1949	Y	1880	376
7/1/1949	Y	1820	364
7/1/1949	Y	1780	356
8/1/1949	Y	2820	564
8/1/1949	Y	1880	376
8/1/1949	Y	2940	588
8/1/1949	Y	1720	344
8/1/1949	Y	1440	288
8/1/1949	Y	2480	496
8/1/1949	Y	1800	360
8/1/1949	Y	2880	576
8/1/1949	Y	1680	336
8/1/1949	Y	2420	484
8/1/1949	Y	2840	568
8/1/1949	Y	2620	524
8/1/1949	Y	2220	444
8/1/1949	Y	2312	462

8/1/1949	Y	2150	430
8/1/1949	Y	2296	459
8/1/1949	Y	1760	352
8/1/1949	Y	1668	334
9/1/1949	Y	2960	592
9/1/1949	Y	520	104
9/1/1949	Y	2780	556
9/1/1949	Y	2320	464
9/1/1949	Y	2420	484
3/26/1963	Y	77	15
3/26/1963	Y	144	29
3/26/1963	Y	78	16
3/26/1963	Y	51	10
5/14/1963	Y	74	15
5/14/1963	Y	86	17
5/14/1963	Y	59	12
6/21/1963	Y	88	18
6/21/1963	Y	177	35
6/21/1963	Y	76	15
6/21/1963	Y	69	14
7/10/1963	Y	359	72
7/10/1963	Y	365	73
7/10/1963	Y	406	81
7/10/1963	Y	153	31
7/22/1963	Y	267	53
7/22/1963	Y	403	81
7/22/1963	Y	48	10
8/8/1963	Y	220	44
8/8/1963	Y	260	52
8/8/1963	Y	272	54
8/8/1963	Y	106	21
8/22/1963	Y	213	43
9/4/1963	Y	291	58
9/4/1963	Y	226	45
9/4/1963	Y	164	33
9/4/1963	Y	92	18
9/17/1963	Y	230	46
9/17/1963	Y	293	59
9/17/1963	Y	279	56
9/17/1963	Y	111	22
10/5/1963	Y	211	42

10/5/1963	Y	392	78
10/5/1963	Y	268	54
10/5/1963	Y	34	7
11/2/1963	Y	454	91
11/2/1963	Y	241	48
11/2/1963	Y	462	92
11/2/1963	Y	98	20
12/1/1963	Y	216	43
12/1/1963	Y	424	85
12/1/1963	Y	243	49
12/1/1963	Y	74	15
1/1/1964	Y	30	6
1/1/1964	N	0	0
1/1/1964	Y	23	5
1/1/1964	Y	77	15
1/1/1964	Y	76	15
2/1/1964	Y	16	3
2/1/1964	N	0	0
2/1/1964	Y	58	12
2/1/1964	Y	45	9
2/1/1964	Y	41	8
3/1/1964	Y	16	3
3/1/1964	N	0	0
3/1/1964	Y	45	9
3/1/1964	Y	41	8
3/1/1964	Y	39	8
4/1/1964	Y	13	3
4/1/1964	Y	13	3
4/1/1964	Y	48	10
4/1/1964	Y	38	8
4/1/1964	Y	15	3
5/1/1964	Y	19	4
5/1/1964	Y	9	2
5/1/1964	Y	70	14
5/1/1964	Y	38	8
5/1/1964	Y	106	21
6/1/1964	Y	28	6
6/1/1964	Y	21	4
6/1/1964	Y	9	2
6/1/1964	Y	7	1
6/1/1964	Y	52	10

6/1/1964	Y	66	13
6/1/1964	Y	85	17
6/1/1964	Y	40	8
6/1/1964	Y	31	6
6/1/1964	Y	35	7
7/1/1964	Y	22	4
7/1/1964	Y	20	4
7/1/1964	Y	7	1
7/1/1964	Y	6	1
7/1/1964	Y	60	12
7/1/1964	Y	49	10
7/1/1964	Y	41	8
7/1/1964	Y	73	15
7/1/1964	Y	38	8
7/1/1964	Y	26	5
8/1/1964	Y	22	4
8/1/1964	Y	9	2
8/1/1964	Y	47	9
8/1/1964	Y	71	14
8/1/1964	Y	52	10
9/1/1964	Y	20	4
9/1/1964	Y	7	1
9/1/1964	Y	30	6
9/1/1964	Y	101	20
9/1/1964	Y	43	9
10/1/1964	Y	13	3
10/1/1964	N	0	0
10/1/1964	Y	37	7
10/1/1964	Y	65	13
10/1/1964	Y	58	12
11/1/1964	Y	13	3
11/1/1964	N	0	0
11/1/1964	Y	31	6
11/1/1964	Y	45	9
11/1/1964	Y	48	10
12/1/1964	Y	13	3
12/1/1964	N	0	0
12/1/1964	N	0	0
12/1/1964	N	0	0
12/1/1964	N	0	0
1/1/1965	Y	13	3

1/1/1965	Y	7	1
1/1/1965	Y	26	5
1/1/1965	Y	90	18
1/1/1965	Y	39	8
2/1/1965	Y	12	2
2/1/1965	Y	6	1
2/1/1965	Y	29	6
2/1/1965	Y	87	17
2/1/1965	Y	50	10

Table S3. The time series of toxicity in whole clams obtained from 1948-1949 [9]and 1963-1965 [10]. Toxicity was determined by mouse bioassay and converted to µg STX-eq. 100 g tissue^{-1} as described in the material and methods section.
