# Supplementary material for: Paralytic Shellfish Toxins in Alaskan Butter Clams: Does Cleaning Make Them Safe to Eat?
Source: Toxins (Basel). 2025 May 28;17(6):271. doi: 10.3390/toxins17060271 (PMC12197486; doi:10.3390/toxins17060271)
Supplement: Supplementary file 1 [file toxins-17-00271-s001.zip › Supplementary Table S4.pdf]

Supplementary Table S4

Method	Sample ID	Date Sampled	Replicate number	Number individuals assayed	Body part analyzed	Edible µg STX-eq. /100 g tissue from HPLC	mass (g)	Sample ID	Body part analyzed	Non-edible µg STX-eq. /100 g tissue from HPLC	mass (g)	Whole clam mass (g)	Whole clam µg STX-eq. /100 g tissue from HPLC	%reduction relative to whole clams	Island	City	Collection Beach
1	NPRB464	4/17/2018	1	12	edible	62.3	175.0	NPRB465	non-edible	107.1	57.1	232.1	93.4	33.3	Kodiak Island	Kodiak	Mission Beach
1	NPRB466	4/17/2018	2	12	edible	54.6	159.6	NPRB467	non-edible	94.2	59.0	218.6	83.2	34.4	Kodiak Island	Kodiak	Mission Beach
1	NPRB462	4/17/2018	3	12	edible	58.8	175.3	NPRB463	non-edible	84.0	68.4	243.7	77.0	23.7	Kodiak Island	Kodiak	Mission Beach
1	NPRB588	5/18/2018	1	12	edible	12.3	155.4	NPRB589	non-edible	64.0	66.5	221.9	37.8	67.4	Kodiak Island	Kodiak	Mission Beach
1	NPRB584	5/18/2018	2	12	edible	13.7	157.8	NPRB585	non-edible	59.1	61.4	219.2	37.1	63.1	Kodiak Island	Kodiak	Mission Beach
1	NPRB586	5/18/2018	3	12	edible	15.3	155.6	NPRB587	non-edible	56.6	56.6	212.2	38.2	59.9	Kodiak Island	Kodiak	Mission Beach
1	NPRB603	6/18/2018	1	12	edible	117.9	159.0	NPRB606	non-edible	1189.0	66.6	225.6	610.4	80.7	Kodiak Island	Kodiak	Mission Beach
1	NPRB604	6/18/2018	2	12	edible	97.6	166.1	NPRB607	non-edible	1224.0	60.8	226.9	611.2	84.0	Kodiak Island	Kodiak	Mission Beach
1	NPRB605	6/18/2018	3	12	edible	90.5	149.3	NPRB608	non-edible	887.8	62.5	211.8	483.3	81.3	Kodiak Island	Kodiak	Mission Beach
1	NPRB626	7/17/2018	1	12	edible	43.2	157.7	NPRB629	non-edible	333.0	56.8	214.5	187.3	76.9	Kodiak Island	Kodiak	Mission Beach
1	NPRB628	7/17/2018	2	12	edible	66.6	145.5	NPRB624	non-edible	337.1	55.2	200.7	216.5	69.2	Kodiak Island	Kodiak	Mission Beach
1	NPRB627	7/17/2018	3	12	edible	53.7	150.7	NPRB625	non-edible	209.4	52.5	203.2	143.1	62.5	Kodiak Island	Kodiak	Mission Beach
2	NPRB456	4/19/2018	1	12	edible	90.6	100.7	NPRB457	non-edible	156.7	97.2	197.9	125.8	28.0	Kodiak Island	Old Harbor	Shipwreck Beach
2	NPRB458	4/19/2018	2	12	edible	79.6	129.2	NPRB459	non-edible	143.8	128.3	257.5	96.3	17.3	Kodiak Island	Old Harbor	Shipwreck Beach
2	NPRB460	4/19/2018	3	12	edible	91.0	101.0	NPRB461	non-edible	139.5	94.9	195.9	118.6	23.3	Kodiak Island	Old Harbor	Shipwreck Beach
2	NPRB580	5/18/2018	1	12	edible	13.1	132.8	NPRB581	non-edible	117.3	115.4	248.2	54.7	76.1	Kodiak Island	Old Harbor	Shipwreck Beach
2	NPRB582	5/18/2018	2	12	edible	39.0	140.2	NPRB583	non-edible	124.1	110.4	250.6	71.8	45.7	Kodiak Island	Old Harbor	Shipwreck Beach
2	NPRB578	5/18/2018	3	12	edible	63.0	115.1	NPRB579	non-edible	96.8	112.9	228.0	74.8	15.7	Kodiak Island	Old Harbor	Shipwreck Beach
2	NPRB574	6/18/2018	1	12	edible	349.1	114.8	NPRB575	non-edible	679.6	97.95	212.8	508.3	31.3	Kodiak Island	Old Harbor	Shipwreck Beach
2	NPRB572	6/18/2018	2	12	edible	101.0	126.2	NPRB573	non-edible	534.2	113.9	240.1	276.1	63.4	Kodiak Island	Old Harbor	Shipwreck Beach
2	NPRB576	6/18/2018	3	12	edible	125.6	117.1	NPRB577	non-edible	839.3	113.2	230.3	428.8	70.7	Kodiak Island	Old Harbor	Shipwreck Beach
2	NPRB564	7/16/2018	1	12	edible	49.8	90.8	NPRB565	non-edible	786.9	98.9	189.7	439.2	88.7	Kodiak Island	Old Harbor	Shipwreck Beach
2	NPRB568	7/16/2018	2	12	edible	42.8	101.4	NPRB569	non-edible	587.4	77.2	178.6	353.6	87.9	Kodiak Island	Old Harbor	Shipwreck Beach
2	NPRB566	7/16/2018	3	12	edible	151.0	93.1	NPRB567	non-edible	879.9	78.2	171.3	596.2	74.7	Kodiak Island	Old Harbor	Shipwreck Beach
2	NPRB781	8/15/2018	1	15	edible	93.8	114.7	NPRB782	non-edible	189.7	140.1	254.8	117.2	20.0	Kodiak Island	Old Harbor	Shipwreck Beach
2	NPRB779	8/15/2018	2	15	edible	120.6	106.9	NPRB780	non-edible	302.5	126.1	233.0	185.7	35.1	Kodiak Island	Old Harbor	Shipwreck Beach

2	NPRB777	8/15/2018	3	15	edible	98.6	92.0	NPRB778	non-edible	200.3	97.7	189.7	153.9	35.9	Kodiak Island	Old Harbor	Shipwreck Beach
2	NPRB741	9/19/2018	1	15	edible	94.2	66.0	NPRB742	non-edible	131.8	72.7	138.7	140.4	32.9	Kodiak Island	Old Harbor	Shipwreck Beach
2	NPRB746	9/19/2018	2	15	edible	57.7	83.4	NPRB747	non-edible	127.1	98.9	182.3	96.7	40.3	Kodiak Island	Old Harbor	Shipwreck Beach
2	NPRB743	9/19/2018	3	15	edible	67.3	94.3	NPRB744	non-edible	149.8	102.6	196.9	108.8	38.2	Kodiak Island	Old Harbor	Shipwreck Beach
2	NPRB773	10/30/2018	1	12	edible	65.2	73.1	NPRB773	non-edible	119.2	72.8	145.9	114.9	43.2	Kodiak Island	Old Harbor	Shipwreck Beach
2	NPRB770	10/30/2018	2	12	edible	85.4	76.6	NPRB771	non-edible	158.9	84.7	161.3	139.6	38.8	Kodiak Island	Old Harbor	Shipwreck Beach
2	NPRB775	10/30/2018	3	12	edible	96.1	57.4	NPRB776	non-edible	126.7	84.8	142.2	128.5	25.2	Kodiak Island	Old Harbor	Shipwreck Beach

Table S4. Butter clam toxicity data for whole clams, edible tissue, and non-edible tissue obtained using methods practiced in native communities on Kodiak Island, AK. Edible tissue consists of the body and various amounts of the siphon (See Materials and Methods sections for details). Non-edible tissues consist of gut, siphon black tip and various amounts of the siphon.
